# The Value of Condensed Milk

**Published:** 1906-04-21

**Authors:** John E. Sandilands


### Editor's Letter-Box.

Our Correspondents are reminded that prolixity is a great bar to publication, and that brevity of style and conciseness of statement greatly facilitate early insertion.]

### THE VALUE OF CONDENSED MILK.

The Nestlé and Anglo-Swiss Condensed Milk Company have forwarded to us the following letter from Dr. J. E. Sandilands, with reference to an article on "The Causation of Epidemic Diarrhœa," which appeared in The Hospital of February 24, 1906 :—

Dear Sirs,—I regret that you should feel that the readers of a paper so widely known as The Hospital might misinterpret certain references to your milk, which I originally published in a scientific journal with a limited circulation, and which you now inform me have been mentioned in The Hospital of February 24. My remarks have been misinterpreted if those who have read them do not see that I used your name as a guarantee that the infants to whom I referred were being fed on the best brand of condensed milk in the market. It is a matter of common knowledge that your milk contains all the cream and consequently gives an excellent percentage of fat.

I am no opponent of good condensed milk for infants who cannot obtain or cannot digest good cows' milk, and I believe your particular brand to be a food of great value to the infants of the poorer classes. It is recommended and used by medical men with the greatest benefit for certain cases of digestive disorders in babies, and I have seen the infants of the poor thrive on it.

Knowing the care you take to provide a pure milk and to keep abreast of the medical opinion of the day, I hope you will not take it amiss if I again express my very decided opinion that it is essential that opened tins of milk should be protected from contamination by flies and other agencies during the summer months, and that it would be a great benefit to the public if you were to issue directions to this effect with each tin.

During the South African campaign your milk was used almost universally, and it was the custom to open the tins by drilling one small hole in the centre of the lid and to squeeze the milk out by pressing on the bottom of the tins with the thumbs. After the desired quantity of milk had been expressed the top of the tin was protected from dust and flies by an inverted saucer.

If you would care to do so you are at liberty to ask the Editor of The Hospital to publish this letter as a comment on my own article. I am, Sirs,

Yours faithfully,

John E. Sandilands, M.D.

